# Baseline fat fraction is a strong predictor of disease progression in Becker muscular dystrophy

**DOI:** 10.1002/nbm.4691

**Published:** 2022-02-05

**Authors:** Thom T. J. Veeger, Nienke M. van de Velde, Kevin R. Keene, Erik H. Niks, Melissa T. Hooijmans, Andrew G. Webb, Jurriaan H. de Groot, Hermien E. Kan

**Affiliations:** ^1^ C. J. Gorter Center for High Field MRI, Department of Radiology Leiden University Medical Center (LUMC) Leiden The Netherlands; ^2^ Department of Neurology, Leiden University Medical Center (LUMC) Leiden The Netherlands; ^3^ Duchenne Center Netherlands The Netherlands; ^4^ Department of Radiology & Nuclear Medicine Amsterdam University Medical Centers Amsterdam The Netherlands; ^5^ Department of Rehabilitation Medicine, Leiden University Medical Center (LUMC) Leiden The Netherlands

**Keywords:** Dixon, mixed‐effects model, MRI, MRS, muscle degeneration/disease progression

## Abstract

In Becker muscular dystrophy (BMD), muscle weakness progresses relatively slowly, with a highly variable rate among patients. This complicates clinical trials, as clinically relevant changes are difficult to capture within the typical duration of a trial. Therefore, predictors for disease progression are needed. We assessed if temporal increase of fat fraction (FF) in BMD follows a sigmoidal trajectory and whether fat fraction at baseline (FFbase) could therefore predict FF increase after 2 years (ΔFF). Thereafter, for two different MR‐based parameters, we tested the additional predictive value to FFbase. We used 3‐T Dixon data from the upper and lower leg, and multiecho spin‐echo MRI and 7‐T ^31^P MRS datasets from the lower leg, acquired in 24 BMD patients (age: 41.4 [SD 12.8] years). We assessed the pattern of increase in FF using mixed‐effects modelling. Subsequently, we tested if indicators of muscle damage like standard deviation in water T_2_ (stdT_2_) and the phosphodiester (PDE) over ATP ratio at baseline had additional value to FFbase for predicting ∆FF. The association between FFbase and ΔFF was described by the derivative of a sigmoid function and resulted in a peak ΔFF around 0.45 FFbase (fourth‐order polynomial term: t = 3.7, *p* < .001). StdT_2_ and PDE/ATP were not significantly associated with ∆FF if FFbase was included in the model. The relationship between FFbase and ∆FF suggests a sigmoidal trajectory of the increase in FF over time in BMD, similar to that described for Duchenne muscular dystrophy. Our results can be used to identify muscles (or patients) that are in the fast progressing stage of the disease, thereby facilitating the conduct of clinical trials.

## Graphical abstract

We found a sigmoidal temporal progression of fat fraction (FF) in lower extremity muscles of Becker muscular dystrophy patients, evidenced by the bell‐shaped relationship between baseline FF (FFbase) and FF increase (∆FF). This can be used to identify muscles that are in the fast progressing stage of the disease, thereby facilitating the conduct of clinical trials. Neither baseline standard deviation T_2_ (stdT_2_) nor phosphodiester (PDE) over ATP ratio, markers for muscle cell/membrane damage, were associated with ∆FF.

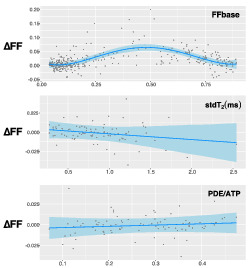

**Abbreviations used**
∆FF: fat fraction increase at 2‐year follow‐up
BMD: Becker muscular dystrophy
CSI: chemical shift imaging
DMD: Duchenne muscular dystrophy
EPG: extended phase graph
FF: fat fraction
FFbase: baseline fat fraction
FSHD: facioscapulohumeral dystrophy
GNE: UDP‐N‐acetylglucosamine 2‐epimerase/N‐acetylmannosamine kinase
MEM: mixed‐effects model
MESE: multi‐echo spin‐echo
PDE/ATP: phosphodiester over ATP ratio
PROM: patient‐reported outcome measure
ROI: region of interest
SI: signal intensity
stdT_2_
: standard deviation T_2_

## 1 INTRODUCTION

Becker muscular dystrophy (BMD) is caused by mutations in the *DMD*‐gene, leading to truncated and reduced levels of the dystrophin protein in muscle.^1^, ^2^ The disease is characterised by variable and progressive muscle weakness, where muscle tissue is replaced by fat and fibrotic tissue.^3^ The disease progression is relatively slow and highly variable among patients, ranging from patients showing severe symptoms at paediatric age up to cases with mild symptoms in the elderly.^4^, ^5^

Currently, there is no cure for the disease, but there are several ongoing clinical trials (NCT‐03238235, NCT‐04585464, NCT‐04386304, NCT‐03879304 and NCT‐04054375). The slow progression and high variability in symptom onset and progression leads to difficulties in the design of such trials, because these limit the chances of observing clinically relevant changes in disease progression within the typical trial duration of 1–2 years.

Muscle fat fraction, measured by quantitative MRI (qMRI) or MRS, is considered as a potential surrogate endpoint in clinical trials in muscular dystrophies.^6^, ^7^ qMRI has been shown to noninvasively, objectively and accurately assess muscle fat fraction with high reproducibility in many muscular dystrophies, including BMD.^8^, ^9^, ^10^ Moreover, fat fraction correlates with function,^11^, ^12^ can predict future loss of function and clinical milestones in Duchenne muscular dystrophy (DMD),^13^, ^14^, ^15^, ^16^ and can potentially capture changes using much smaller sample sizes compared with functional tests.^15^, ^17^ In BMD, the average increase in fat fraction over 2 years is around 0.7%–1.9% for the lower and upper leg,^18^ respectively, which is much lower compared with the 3%–7% over 1 year in DMD patients.^15^ In addition, BMD shows considerable variability between patients with fat fraction increases after 2 years ranging from −1.0% to 6.4%.^18^, ^19^ With these limited changes and large variability, it is very important to be able to identify key muscles or patients that are likely to show a large increase in fat fraction within the duration of a clinical trial.

There are several candidate MR parameters that could be used to predict fat fraction increase over time, including baseline (at the start of measurement) fat fraction, phosphodiester over ATP ratio (PDE/ATP) obtained from ^31^P MRS,^20^ tissue pH,^21^ the average water T_2_‐relaxation time^22^, ^23^ and standard deviation of water T_2_ (stdT_2_).^24^ The fat fraction in DMD follows a sigmoidal trajectory with age,^13^, ^14^ which means that the rate of disease progression is not constant over time, but is highest around the middle region of the fat fraction range. As a result of this trajectory, baseline fat fraction is a good predictor for the rate of fat fraction increase over time in DMD. We recently showed that the largest increases in fat fraction over time occurred in BMD patients with a baseline fat fraction of 40%–60%.^18^ Therefore, we hypothesise that the fat fraction increase over time in BMD follows the same pattern as in DMD and that baseline fat fraction can therefore predict fat fraction increase. PDE/ATP,^20^ tissue pH,^21^ average water T_2_ ^22^, ^23^ and stdT_2_ ^24^ are related to muscle cell/membrane damage and may precede an increase in fat fraction. For instance, in patients with Pompe's disease or GNE (UDP‐N‐acetylglucosamine 2‐epimerase/N‐acetylmannosamine kinase) myopathy, higher baseline water T_2_ values were positively correlated with larger increases in fat fraction after 1 year.^25^, ^26^

In this study, we assessed whether the increase in fat fraction in BMD indeed follows a sigmoidal trajectory and that baseline fat fraction can therefore predict the amount of fat replacement in 2 years. Subsequently, we assessed if measures for muscle damage had additional predictive value to baseline fat fraction for the progression of fat replacement after 2 years. To limit the amount of predictors, we only selected PDE/ATP and stdT_2_, as these have been shown to be different between BMD patients compared with healthy controls.^27^ We chose to include stdT_2_ and not average water T_2_ because muscle tissue is most likely not replaced by fat at the same rate throughout the muscle.^18^ If only parts of the muscle show higher T_2_ values, resulting in larger std values for T_2_, average T_2_ might not be as sensitive to subsequent change in fat fraction.

## 2 METHODS

### 2.1 Subjects

The MRI and MRS datasets from BMD patients used in this study were previously described by Hooijmans et al.^27^ and van de Velde et al.^18^ Briefly, male BMD patients were scanned at baseline and again after about 2 years. Patients were recruited from the Dutch Dystrophinopathy Database and diagnosis was confirmed by genetic testing.^28^ The study was approved by the local medical ethical committee and written informed consent was given by all patients.

### 2.2 MR acquisition

The MRI examinations were performed at 3 and 7 T, as reported in detail by Hooijmans et al.^27^ and van de Velde et al.^18^ Briefly, on the 3‐T MR scanner (Ingenia, Philips Healthcare, Best, The Netherlands), scans included a three‐point gradient echo Dixon sequence (T_R_/T_E_/ΔT_E_ 210/4.41/0.76 ms; NSA 2; flip angle 8°; voxel size 1 x 1 x 10 mm^3^; slice gap 5 mm; 23 slices) to quantify muscle fat replacement in the upper and lower leg muscles and a multi‐echo spin‐echo (MESE) multislice sequence (17 echoes; T_R_/T_E_/ΔT_E_ 3000/8/8 ms; voxel size 1.4 x 1.8 x 10 mm^3^; slice gap 20 mm; five slices) to assess water T_2_‐relaxation times in the lower leg. Upper leg scans were aligned perpendicular to the femur and lower leg scans to the tibia. On the same day, ^31^P MRS examinations were performed on a 7‐T MR scanner (Achieva, Philips Healthcare, Best, The Netherlands) and included a ^31^P 2D chemical shift imaging (CSI) scan to asses phosphorus metabolites in the lower leg. This scan was positioned in such a way that one individual voxel was located within a single muscle over the full length of the coil. The 3‐T scans were performed using a 16‐element receive coil placed on the leg and a 12‐element coil in the table under the leg and 7‐T scans using a custom‐built double‐tuned (^31^P and ^1^H) volume coil around the leg.

### 2.3 MRI analysis

Water and fat images were reconstructed offline in Matlab 2019b (MathWorks, Natick, MA) from the 3‐T Dixon data using an in‐house water‐fat separation algorithm, described in detail by Hooijmans et al.^27^ This algorithm is based on a six‐peak lipid spectrum.^29^ Water T_2_‐relaxation times were calculated from the MESE data using the approach described by Keene et al.^30^ Briefly, an extended phase graph (EPG) fitting approach was used, which considered different relaxation times for a single water and a single fat component, and which were fitted with a dictionary method on a voxel‐by‐voxel basis. This dictionary was created using water T_2_ values from 10 to 60 ms. Voxels that fitted on the boundaries of the dictionary were excluded because they could not be physiologically correct.

**FIGURE 1:**
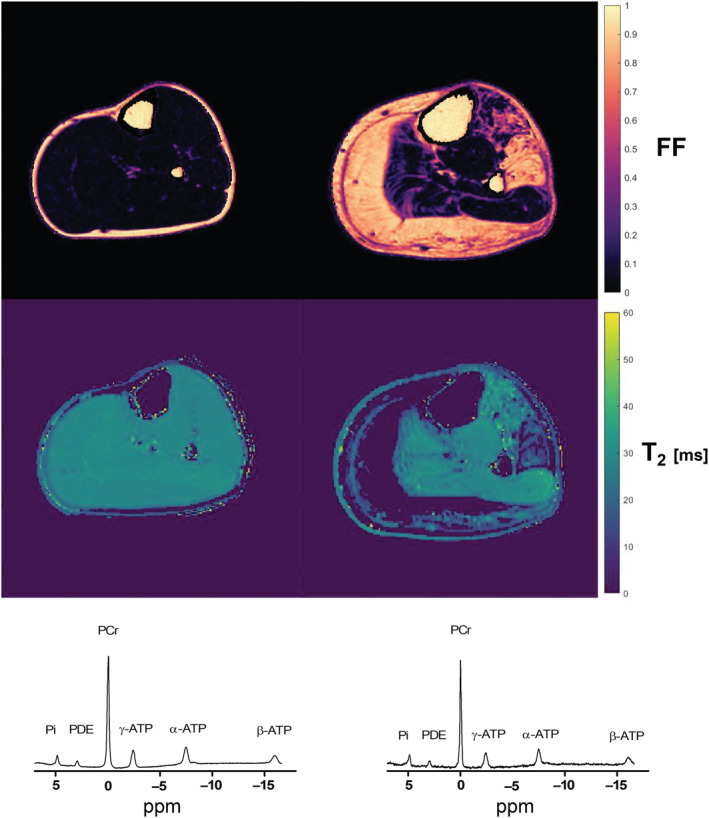
Representative fat fraction (upper row), water T_2_ maps (middle row) and MRS spectra of the soleus (lower row) for a patient in a relatively early (left column) and relatively late (right column) stage of the disease. For the water T_2_ maps, voxels that fitted on the boundaries of the dictionary (i.e., 0 and 60 ms) were excluded, labelled as 0 in these maps. The MRS spectra were fitted in the time domain using Gaussian line shapes and prior knowledge of the linewidths of the PDE and ß‐ATP.^27^ FF, fat fraction; PCr, phosphocreatine; PDE, phosphodiester; Pi, inorganic phosphate; ppm, parts per million

Images were visually evaluated for bulk motion artefacts and were excluded if these were present. Regions of interest (ROIs) were drawn on the borders of the muscles in five slices for six lower leg muscles and for 10 upper leg muscles using the Medical Image Processing, Analysis and Visualization program (http://mipav.cit.nih.gov). The six lower leg muscles consisted of gastrocnemius medialis, gastrocnemius lateralis, soleus, tibialis anterior, tibialis posterior and peroneus. The 10 upper leg muscles were rectus femoris, vastus medialis, vastus lateralis, vastus intermedius, biceps femoris long head, semitendinosus, semimembranosus, adductor magnus, gracilis and sartorius. The slice containing the insertion of the flexor digitorum longus muscle was chosen to be the centre for the lower leg slices. The middle slice for the upper leg was defined as the most proximal slice where the short head of the biceps femoris was still visible. Because the slice gap was larger for the T_2_ images, the middle three slices were used for further analyses, which coincided with the middle and outer slices of the five analysed Dixon slices. ROIs drawn on the Dixon image were eroded by two voxels and the ones drawn on the T_2_ image were eroded by one voxel, because of the larger voxel size of the T_2_ acquisition. Fat replacement per muscle and per slice was quantified by the fat fraction quantified by signal intensity (SI) fat over the summed SI of fat and water: SI fat/(SI fat + SI water) and averaged per ROI. From the T_2_ maps, the standard deviation of T_2_ was calculated per ROI to get a stdT_2_ per muscle and per slice. ROIs with less than 50% remaining voxels, due to the exclusion of voxels fitting on the physiological boundaries of the dictionary, were excluded. Subsequently, an area‐weighted average fat fraction and stdT_2_ of the five and three slices, respectively, were calculated per muscle. Representative fat fraction and water T_2_ maps for two patients in different stages of the disease, reflected by the amount of fat replacement, are shown in Figure 1.

### 2.4 MRS analysis

The MRS analysis was previously reported by Hooijmans et al.^27^ Briefly, the ^31^P MRS datasets were exported as free induction decays and processed in the time domain using AMARES in the JMRUI software package (version 5; http://sermn02.uab.es/mrui/).^31^ All signals were fitted using Gaussian line shapes and the signal of PDE was presented as the ratio over γ‐ATP. Representative spectra for two patients in different stages of the disease, as indicated by the amount of fat replacement, are shown in Figure 1.

### 2.5 Statistical analysis

A mixed‐effects model 1 (MEM1) was used to determine the pattern of temporal progression of fat fraction in BMD. We hypothesised that the association between baseline fat fraction (FFbase) and the increase in fat fraction at the 2‐year follow‐up (∆FF) would follow the trajectory of the derivative of a sigmoidal function, a bell‐shaped curve. To be able to capture this relationship, with four distinguishably different slopes (zero – positive – negative – zero), up to the fourth (orthogonal) polynomial term for the association between FFbase and ΔFF was added to the model. The model also included the first three polynomial terms as a fourth‐order includes all four polynomial terms. For every order we assessed if the fit was significantly better when that order was added to the previous ones. Because in BMD proximal muscles in general are involved earlier than distal muscles,^32^ both upper and lower leg fat fraction data were used to include data over a larger range of FFbase. To account for varying disease onset between patients and muscles, a by‐muscle and by‐patient random intercept was added to the model.

In mixed‐effects model 2 (MEM2), we assessed if measures for muscle membrane damage (stdT_2_ and PDE/ATP) had additional predictive value to FFbase for ΔFF. This model only included lower leg data with ΔFF as an outcome variable, and FFbase, stdT_2_ and PDE/ATP as predictor variables, because no T_2_ and ^31^P data were available for the upper leg. For the association between FFbase and ∆FF, up to the fourth (orthogonal) order polynomial was added for the association between FFbase and ∆FF (in accordance with the outcome of MEM1). To account for varying disease onset between patients and muscles, a by‐muscle and by‐patient random intercept was added to the model.

**FIGURE 2:**
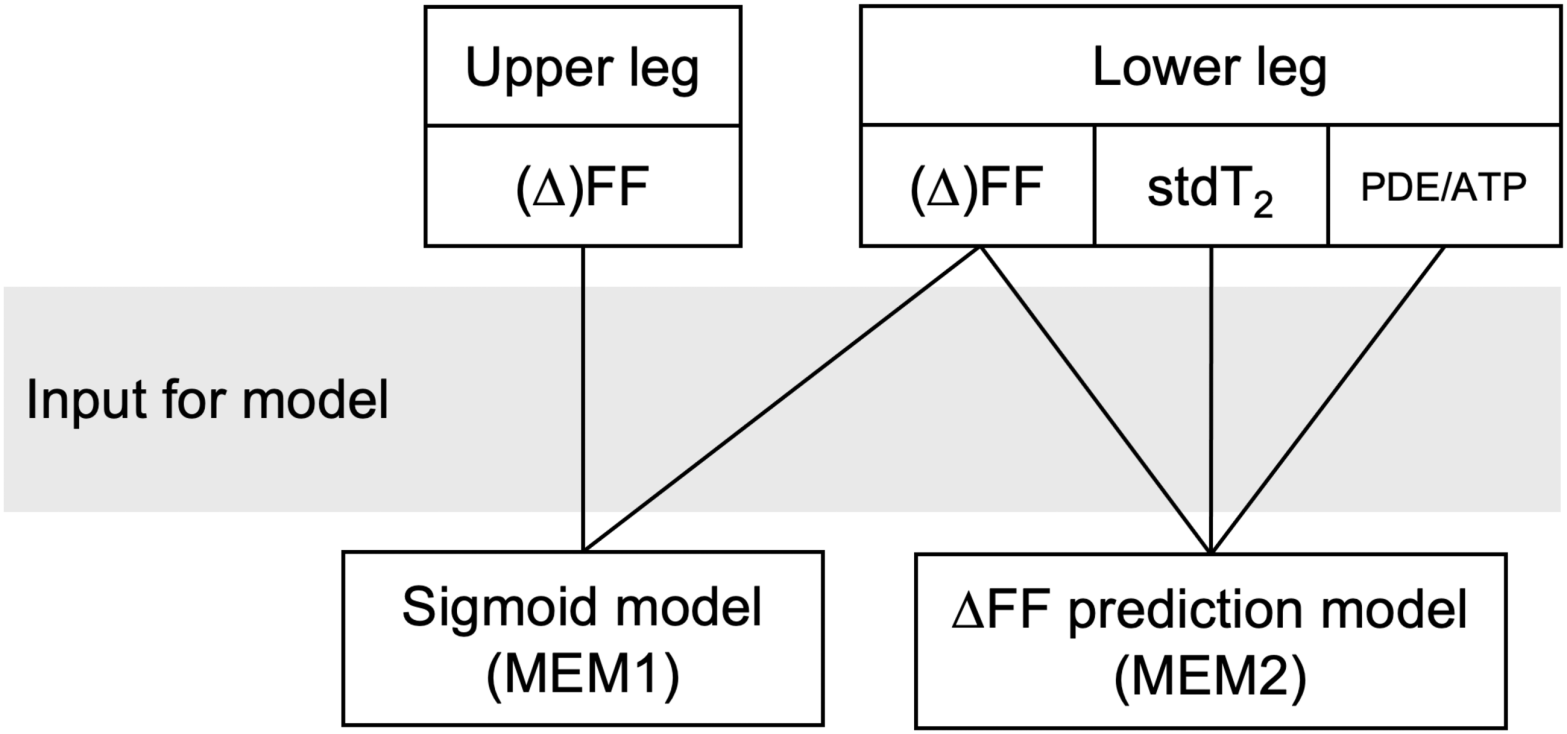
A schematic representation of the workflow of the statistical analysis. The upper two rows indicate the type of data available for the upper and lower leg: fat fraction (FF), standard deviation of water T_2_ (stdT_2_) and phosphodiester over ATP (PDE/ATP). The third row refers to the two models and the lines indicate in which models the data were included

A schematic representation of the workflow can be found in Figure 2. All models and graphs were built using the software package R (R Core Team, 2019) in combination with the packages *lme4* ^33^ and *visreg*.^34^ *p* values for the fixed effects were obtained by t‐tests using the *lme4Test* package^35^ and significance was set at *p* less than .05. The full code is publicly available at Github (https://git.lumc.nl/neuroscience/2021-veegerttj-sigmoidal-trajectory-bmd; using the version at commit 466a3b58).

## 3 RESULTS

In total, 24 BMD patients participated in this study (Table 1). Data of four patients were missing for follow‐up due to death (n = 1), implantable cardioverter defibrillator placement (n = 1), scan of the wrong leg (n = 1) and no show (n = 1), and from three patients no usable 7‐T MRS data of the lower leg were acquired, because of quality issues. As a result, upper and lower leg data from 20 patients were included in MEM1, with FFbase as predictor of ∆FF, and lower leg data from 17 patients for MEM2, with FFbase, stdT_2_ and PDE/ATP as predictors of ΔFF.

**TABLE 1:** Descriptive statistics

Patients	Number	24
Age (years)	Mean (SD)	41.4 (12.8)
	Range	18.8–68.2
Length (cm)	Mean (SD)	179.1^*^ (7.6)
Weight (kg)	Mean (SD)	77.0^*^ (12.4)
lost ambulation		
Baseline	Number	0
24 months	Number	1

Abbreviation: SD, standard deviation.

^*^ not available for all patients.

### 3.1 Sigmoidal association between FFbase and ∆FF (MEM1)

**FIGURE 3:**
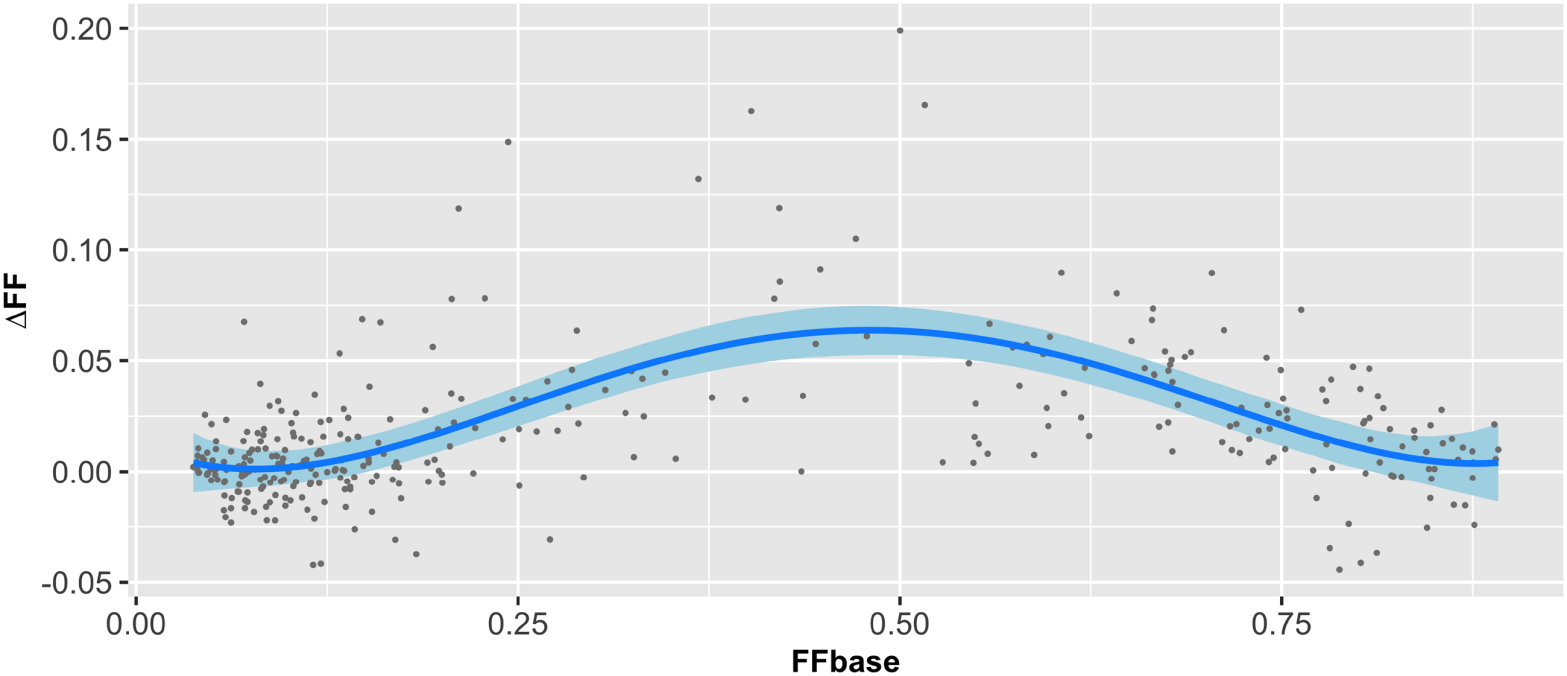
Prediction fit (blue line) and 95% confidence interval (light blue band) for the effect of baseline fat fraction (FFbase) on fat fraction increase at 2‐year follow‐up (∆FF), including a fourth‐order polynomial association. The 340 available datapoints are indicated by the dots. In this analysis, both upper and lower leg data were used

The *p* values in Table 2 indicate whether adding a certain order of the polynomial to the model significantly improved the fit compared with a model only including the previous orders. Thus, the results of MEM1 show that adding the fourth‐order term significantly improved the fit of the association between FFbase and ∆FF (t = 3.709, *p* < .001). The association is visualised in Figure 3, where the fourth‐order polynomial fit is plotted over the data. The fit resulted in a peak ΔFF at around 0.45 FFbase and the lowest ΔFF towards the end of the FFbase range.

**TABLE 2:** Output mixed‐effects model 1

	B (a.u.)	SE B (a.u.)	95% CI (a.u.)	t‐value	*p* value
FFbase – first polynomial	0.139	0.039	0.057–0.217	3.581	**<.001**
FFbase – second polynomial	−0.296	0.031	−0.358 to −0.234	−9.500	**<.0001**
FFbase – third polynomial	−0.060	0.031	−0.120–0.001	−1.941	.053
FFbase – fourth polynomial	0.109	0.029	0.051–0.166	3.709	**<.001**

*Note*: Significant *p* values are presented in bold.

Abbreviation: FFbase, baseline fat fraction.

### 3.2 Predictors for ∆FF (MEM2)

**FIGURE 4:**
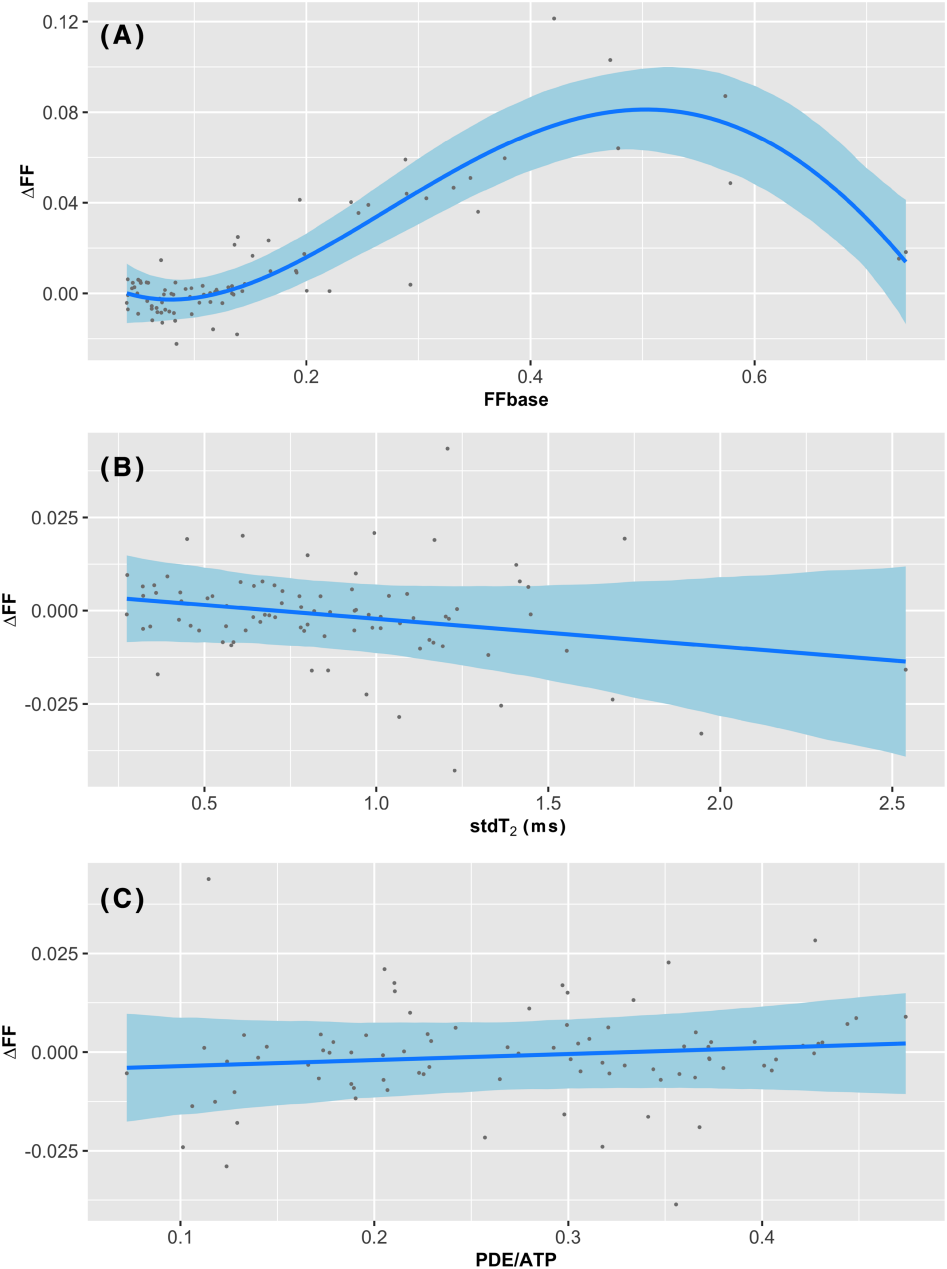
Prediction fits (blue lines) for the three fixed effects, (A) Baseline fat fraction (FFbase), (B) Standard deviation of water T_2_ (stdT_2_) and (C) Phosphodiester over ATP (PDE/ATP), and their effect on fat fraction at 2‐year follow‐up (∆FF), while keeping the other two variables constant at the median value, with the 95% confidence intervals (light blue bands) and the partial residuals for the 86 available datapoints (dots). In this analysis, only lower leg data were used

One datapoint of the stdT_2_ dataset was marked as an outlier (Z‐score = 3.13) and influential point (Cook's distance = 0.50), and was therefore removed from the analysis. The results of MEM2 showed that only FFbase, but neither stdT_2_ nor PDE/ATP, significantly predicted ΔFF (Table 3). When inspecting the relationships in more detail, we observed, in line with MEM1, that ΔFF increased with increasing FFbase up to a value of around 0.5, and that with higher FFbase values ΔFF decreased again (Figure 4A). However, in this model the fourth‐order polynomial term did not reach significance and therefore did not significantly improve the fit compared with a third‐order fit (Table 3). Higher values of stdT_2_ showed slightly lower values of ΔFF, although not significantly (Figure 4B, Table 3), and PDE/ATP clearly showed no relationship with ΔFF (Figure 4C, Table 3).

**TABLE 3:** Output mixed‐effects model 2

	B (a.u.)	SE B (a.u.)	95% CI (a.u.)	t‐value	*p* value
FFbase – first polynomial	0.169	0.027	0.109–0.229	6.161	**<.0001**
FFbase – second polynomial	−0.089	0.019	−0.127 to −0.052	−4.805	**<.0001**
FFbase – third polynomial	−0.092	0.017	−0.127 to −0.057	−5.413	**<.0001**
FFbase – fourth polynomial	0.013	0.015	−0.017–0.044	0.866	.389
stdT_2_ (ms)	−0.007	0.007	−0.021–0.006	−1.123	.265
PDE/ATP	0.015	0.023	−0.032–0.068	0.654	.517

*Note*: Significant *p* values are presented in bold.

Abbreviations: FFbase, baseline fat fraction; stdT_2_, standard deviation T_2_; PDE, phosphodiester.

## 4 DISCUSSION

We used mixed‐effects modelling to test the association between baseline fat fraction and ∆FF at the 2‐year follow‐up in BMD patients. Subsequently, we analysed whether baseline stdT_2_ and PDE/ATP had an additional predictive value to baseline fat fraction when predicting ΔFF. This could help to identify patients or individual muscles that are likely to show a faster progression of the disease within the duration of a clinical trial. Our results showed that the association between baseline fat fraction and ΔFF was described by the derivative of a sigmoid function. Thus, as in DMD, fat replacement of muscles in BMD follows a sigmoidal trajectory. In addition to baseline fat fraction, neither baseline PDE/ATP nor baseline stdT_2_ were significantly associated with ∆FF in BMD patients. The slow disease progression in BMD hampers the design of clinical trials, because this limits the chances of observing clinically relevant changes within the typical duration of a trial of 1–2 years. Therefore, there is a need for parameters that are able to identify patients, or key muscles, that are likely to show a faster progression of the disease within the duration of a clinical trial. Our associations indicate that the largest ΔFF after 2 years can be expected in muscles with baseline fat fraction values of around 0.45. This observation, illustrated by a fourth‐order polynomial relationship between baseline fat fraction and ΔFF, is in line with our hypothesis that in BMD, as in DMD,^13^, ^14^ the temporal progression of fat fraction follows a sigmoidal trajectory.

The existence of this sigmoidal relationship in BMD is an important finding for both designing clinical trials and interpreting their results. In DMD, it was shown to be helpful for the estimation of important disease progression milestones, such as the age of the patients at which individual muscles would reach a certain fat fraction. For the vastus lateralis muscle in DMD, the age of the patient when it reaches a fat fraction of 0.5 strongly correlates with the loss of ambulation.^7^, ^13^, ^14^ Moreover, it means that averaging the progression over all patients or muscles could mask important effects of a drug or intervention, because patients or muscles in different stages of the disease will show a different magnitude of change. Also, when defining inclusion criteria for a clinical trial, one should try to aim for patients or muscles that are in the fast progressing stage of the disease. Unfortunately, due to the rarity of the disease, selecting BMD patients based on disease stage might not always be feasible, even on an international scale. As an alternative, key muscles for every patient individually could be selected and used as an outcome. This does, however, have the disadvantage that a relation to function or physical function patient‐reported outcome measures (PROMs) might be harder to establish.

For baseline stdT_2_, higher values tended towards lower ΔFF values, although not significantly. The individual correlation with ∆FF, without baseline fat fraction taken into account, suggested that higher stdT_2_ values were associated with higher ∆FF (data not shown). This indicates that most of the association between stdT_2_ and ∆FF can be attributed to stdT_2_ being higher with a higher (baseline) fat fraction, which could be explained by lower T_2_ values for muscles with a higher fat fraction.^30^, ^36^

Baseline PDE/ATP did not have significant predictive value over ∆FF. In addition, when PDE/ATP alone was related to ∆FF, no association was found either (data not shown), suggesting that PDE/ATP has no relation to ∆FF in BMD. This is in agreement with the previously found weak correlation between fat fraction and PDE/ATP.^37^ It should be noted that there was a difference in coverage between the MRI and MRS acquisition (7 and 12 cm, respectively). Although the ^31^P coil is indeed 12 cm long, the sensitive region in the long axis is smaller, probably closer to 9 cm, due to the birdcage properties. This results in approximately 0.75 cm extra on both ends of the MRS region. This could result in the inclusion of more fat‐replaced muscle regions in the MRS as a result of proximodistal differences.^18^ Because it is not clear if all muscles will show more fat replacement towards both ends, we expect only small differences over the complete dataset. Given that we found a negligible association between PDE/ATP and ∆FF, we feel that the small difference in coverage will only have had a limited effect, although we cannot exclude it.

Apart from BMD and DMD, a sigmoidal trajectory of the progression of fat fraction is presumably present in other muscular dystrophies as well, even although it was never formally tested. These include facioscapulohumeral muscular dystrophy (FSHD), spinal muscular atrophy, LGMDR9, GNE myopathy and Pompe's disease.^25^, ^38^, ^39^, ^40^, ^41^, ^42^, ^43^ For example, in FSHD patients, muscles with intermediate fat fractions occur much less frequently compared with either high or low fat fractions.^38^, ^39^ This is likely a direct result of a sigmoidal trajectory, because there is a smaller time window to select muscles within the intermediate range and thus a smaller chance of randomly including these muscles. In line with our results, in FSHD and LGMDR9, muscles with baseline fat fraction values in the middle range indeed showed a greater increase in fat fraction,^41^, ^42^ and in GNE myopathy similar results were shown, although this was only tested using a linear association.^43^ By contrast, in Pompe's disease a strong linear relationship between baseline fat fraction and ∆FF was found.^25^ However, data were averaged over all patients for every upper and lower leg muscle, resulting in mean fat fraction values of up to about 50%, meaning that possibly only the ascending linear part of the curve was fitted. It would be interesting to perform similar analyses as described in the current paper in other muscular dystrophies, to test if a sigmoidal trajectory fat fraction progression can be found in other patient populations as well.

In the current study we did not take the age of the patients into account, while it is highly possible that an interaction effect between baseline fat fraction and age exists. In other words, the slope of the S‐curve could be steeper for patients with higher fat fractions at a younger age. Unfortunately, the current dataset contains insufficient individual follow‐up observations to test this hypothesis. In future work this could be investigated by acquiring more longitudinal datapoints per patient to fit individual S‐curves for each muscle. Consecutively, it could be tested if the slope of these S‐curves is associated with the age and disease severity of the patient.

This study has some limitations. First, when only using lower leg data (MEM2), a fourth‐order polynomial was not significantly better than a third‐order fit. This can be explained by the lack of data in the higher range of baseline fat fractions. Including also upper leg data (MEM1) resulted in a significant fourth‐order fit because more datapoints in the higher range of baseline fat fractions were included. Second, a large variance in ΔFF around the peak of the 0.45 baseline fat fraction was observed in both MEM1 and MEM2. This can be explained by the variability in the rate of disease progression between patients and muscles. Baseline fat fraction reflects the stage within the progression when the largest ΔFF can be expected but is not able to predict the amount of ∆FF. In other words, in both slow and fast degenerating muscles, the largest change in fat fraction is expected when the baseline fat fraction is around 0.45.

In conclusion, temporal progression of fat fraction in BMD follows a sigmoidal trajectory because the relationship between baseline fat fraction and the derivative ΔFF could be described by a fourth‐order polynomial. Baseline fat fraction can therefore be used to predict fat fraction increase over 2 years, while baseline stdT_2_ and PDE/ATP did not add significant predictive value. This can be used to identify muscles (or patients) that are in the fast progression stage of the disease.

## CONFLICT OF INTEREST

The Leiden University Medical Center and Amsterdam Medical Center are members of the European Reference Network for Rare Neuromuscular Diseases (ERN EURO‐NMD).
